# Global landscape of yttrium-90 clinical trials: a systematic registry-based analysis

**DOI:** 10.1186/s13063-026-09659-7

**Published:** 2026-03-27

**Authors:** Qianwen Ni, Kenan Wang, Wenyi Jin, Yakun Gao, Ping Gong, Zhenguang Wang, Zheqi Xu, Hui Liu, Susu Luo

**Affiliations:** 1 The Third Department of Hepatic Surgery, Eastern Hepatobiliary Surgery Hospital, 225 Changha Road, Yangpu District, Shanghai, 200438 China; 2 The Proof of Concept Center, Eastern Hepatobiliary Surgery Hospital, Shanghai, 200438 China; 3 Department of Biomedical Sciences, City University of Hong Kong, Hong Kong, 999077 China; 4 Department of Orthopaedics, Renmin Hospital of Wuhan University, Wuhan University, Wuhan, 430060 China; 5 Department of Plastic Surgery, Huashan Hospital, Fudan University, Shanghai, 200120 China; 6 Moores Cancer Center, School of Medicine, University of California San Diego, La Jolla, CA 92122 USA; 7 University of California San Diego, 9500 Gilman Dr., La Jolla, CA 92093 USA

**Keywords:** Yttrium-90, Radioembolization, Clinical trials, Liver cancer, Registry analysis, ClinicalTrials.gov

## Abstract

**Background:**

Yttrium-90 (Y-90) selective internal radiation therapy (SIRT) has emerged as an important locoregional treatment for liver and other malignancies. However, despite its expanding clinical application, the global landscape of Y-90 clinical trials has not been systematically characterized. The objective of this study was to systematically characterize the global portfolio of Y-90 clinical trials, compare liver cancer with non–liver cancer indications, and evaluate patterns of result dissemination.

**Methods:**

We conducted a cross-sectional registry-based analysis of all Y-90 clinical trials registered on ClinicalTrials.gov from 2000 to 2025. Trial characteristics, temporal and geographic patterns, methodological features, and reported outcomes were summarized. Comparative analyses were performed between liver cancer and non–liver cancer trials. Registry entries were linked to PubMed to assess publication output.

**Results:**

A total of 373 eligible trials were identified. Most were interventional (86.9%), treatment-focused (93.1%), and single-center (70.1%), with small sample sizes (54.2% enrolled ≤ 30 participants). Nearly half were phase 1–2 (47.7%) or phase 2–3 (45.8%), with only 6.5% reaching phase 3–4. Randomization was reported in 43.5% of trials, but masking was rare (3.4%). Geographically, studies were concentrated in North America (68.4%), with limited representation from Asia (11.5%) despite its high disease burden. Liver cancer accounted for 44.5% of trials and was more likely than non–liver cancer studies to be randomized, advanced-phase, and Asia-based. Among 93 (24.9%) discontinued trials, accrual difficulties were the leading cause (38.7%). Only 68 trials (18.2%) had published results, with efficacy and safety endpoints more consistently reported in liver cancer studies.

**Conclusions:**

Y-90 research has expanded globally, especially in liver cancer, but trials remain largely small, early phase, and geographically uneven, with limited dissemination. Strengthening methodological rigor through adequately powered multicenter trials, enhancing transparency in result dissemination, and encouraging broader research efforts in high-burden regions such as Asia may help further clarify the optimal role of Y-90 in oncology.

**Supplementary Information:**

The online version contains supplementary material available at 10.1186/s13063-026-09659-7.

## Introduction

Yttrium-90 (Y-90) selective internal radiation therapy (SIRT), also known as transarterial radioembolization (TARE), is a locoregional treatment in which radioactive microspheres are delivered through the hepatic arterial supply to deliver high-dose beta radiation directly to tumors while sparing surrounding parenchyma [1, 2]. Over the past two decades, Y-90 has become an important therapeutic option across a spectrum of diseases, including primary liver cancer, metastatic colorectal cancer, lymphoma, and neuroendocrine tumors [3].

Among these applications, liver cancer, particularly hepatocellular carcinoma (HCC) and intrahepatic cholangiocarcinoma (ICC), has emerged as the predominant indication for Y-90 therapy [2]. Liver cancers represent a major global health burden, ranking as the third leading cause of cancer mortality worldwide, with the highest incidence in Asia [4]. For patients with intermediate or advanced disease who are not eligible for curative surgery or transplantation, Y-90 provides a targeted approach that can achieve tumor downstaging, improve intrahepatic control, and serve as a bridge to transplantation. Notably, the multicenter LEGACY study demonstrated that Y-90 radioembolization achieved high response rates and durable tumor control in patients with solitary, unresectable HCC ≤ 8 cm, with a best overall response rate of 88.3% and three-year overall survival of 86.6% [5]. These findings underscore Y-90’s potential as an effective locoregional therapy, and other clinical studies have further suggested that it may offer comparable or even superior outcomes to transarterial chemoembolization (TACE), with lower toxicity and better quality of life [6–8].

Despite its clinical promise, the research landscape of Y-90 remains fragmented. Most trials are small, single-center, and early phase, limiting the strength of evidence and its incorporation into international guidelines. The majority of studies have been conducted in North America, whereas high-burden regions such as Asia remain underrepresented. Furthermore, many trials face recruitment challenges, high discontinuation rates, and incomplete result dissemination. Cost and accessibility are additional barriers, particularly in low- and middle-income regions. A systematic review of Y-90 radioembolization indicated that the cost of TARE treatment varied widely according to Barcelona Clinic Liver Cancer (BCLC) staging system [9]. Y-90-TARE was associated with lower treatment costs than sorafenib but higher treatment costs when compared to TACE or ablative therapy [9].

To date, no comprehensive study has systematically examined the global portfolio of Y-90 clinical trials, particularly with respect to differences between liver cancer and non–liver cancer indications. Understanding these patterns is essential to identify research gaps, optimize trial methodology, and guide future clinical applications. To address this need, we conducted a cross-sectional registry-based analysis of all Y-90 trials registered on ClinicalTrials.gov from 2000 to 2025. We characterized their temporal and geographic distribution, methodological features, and reported outcomes, and compared studies of liver cancer versus non–liver cancer indications. In addition, we linked registry entries with PubMed to identify trials with published articles, allowing us to evaluate the characteristics of published Y-90 trials.

## Methods

### Study design and data source

This study is a cross-sectional registry-based analysis of clinical trials utilizing Y-90 as a primary treatment. We identified all eligible studies registered on ClinicalTrials.gov between February 29, 2000, and April 29, 2025. All data were extracted as a snapshot on April 29, 2025, and trial inclusion was based on registry records available at that time, regardless of subsequent updates. ClinicalTrials.gov (https://clinicaltrials.gov/), developed by the U.S. National Library of Medicine, is the world’s largest publicly accessible clinical trial database. It serves as a centralized platform for registering and disseminating information on clinical trials across various phases, sponsors, and countries, enhancing research transparency and accountability. This study was conducted in accordance with the Strengthening the Reporting of Observational Studies in Epidemiology (STROBE) guidelines to support methodological clarity and comprehensive reporting [10].

### Search strategy

Two search strategies were applied. The first was a basic search using the terms: “yttrium 90”, “Yttrium-90”, “90Y”, “Y-90”, or “yttrium Y 90.” The second was an expert search using the advanced query: AREA[OfficialTitle] (yttrium 90) OR AREA[BriefTitle] (yttrium 90) OR AREA[InterventionName] (yttrium 90) OR AREA[DetailedDescription] (yttrium 90) OR AREA[BriefSummary] (yttrium 90). Both approaches returned identical results, yielding a total of 416 studies. This study focused on trials evaluating Y-90 as a therapeutic agent, including those involving Y-90-binding antibodies. Studies involving other radioactive elements with similar properties to Y-90, which only mentioned Y-90 in the summary or detailed description, were excluded. In addition, studies referencing the chemical elements “Y” and the number “90” in unrelated contexts were also excluded.

**Fig. 1:**
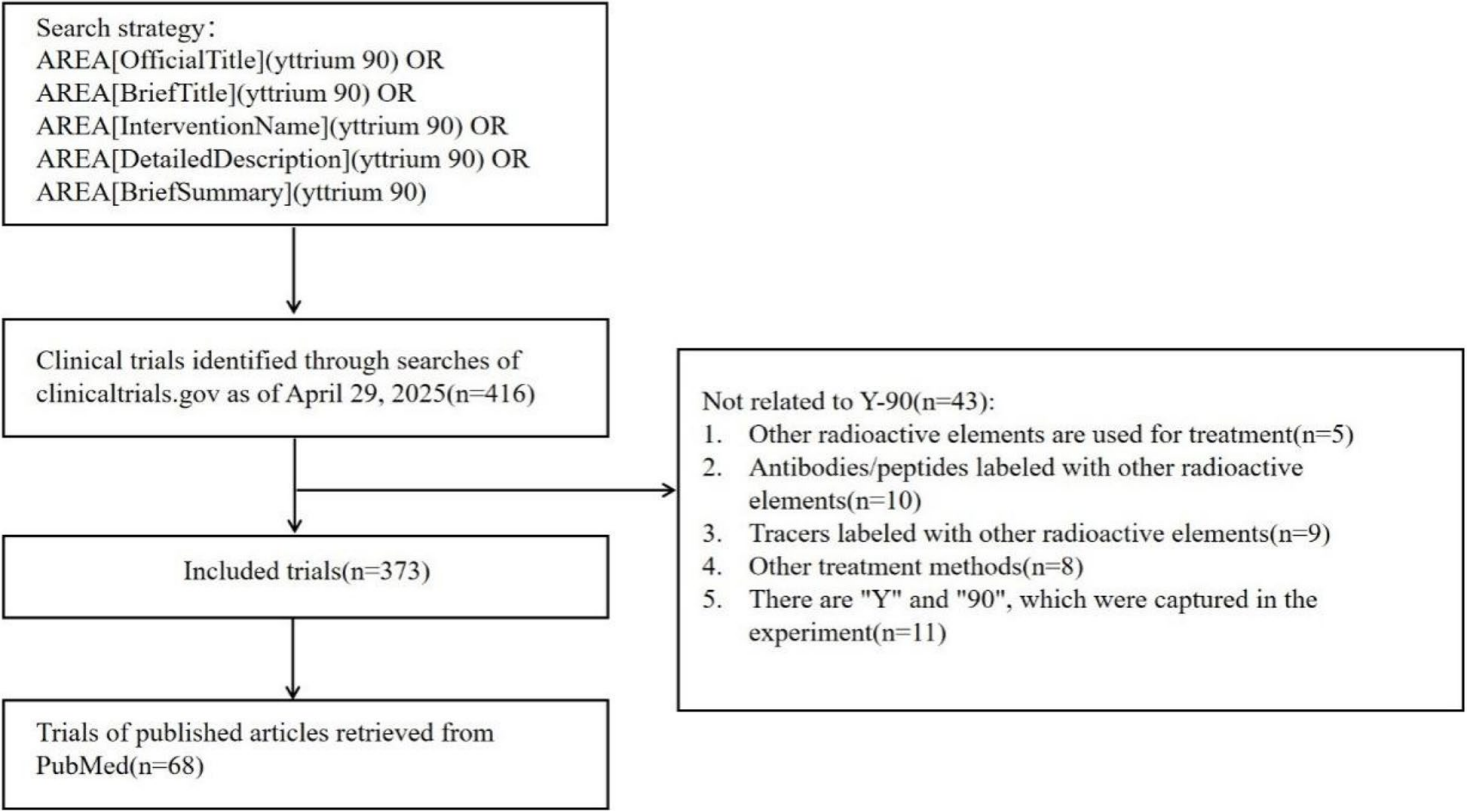
Flowchart of study selection for Yttrium-90 related clinical trails. Through the expert search, the search was conducted on Clinicaltrials.gov by using the search formula, and a total of 416 Y90-related clinical trials were retrieved as of April 29, 2025. Then, through manual screening, 43 studies unrelated to Y90 were excluded, resulting in 373 clinical trials. Furthermore, a literature association search was performed on these 373 trials and the PubMed database, and 68 trials with published research articles were screened out. The query statement for the expert search is: AREA[OfficialTitle] (yttrium 90) OR AREA[BriefTitle] (yttrium 90) OR AREA[InterventionName] (yttrium 90) OR AREA[DetailedDescription] (yttrium 90) OR AREA[BriefSummary] (yttrium 90). Tests involving other radioactive elements with similar properties to Y-90; and studies that only mention Y-90 in the abstract or detailed description; tests that mention the chemical element "Y" and the number "90" in an unrelated context are excluded

After systematic screening based on predefined criteria, 43 studies were excluded, resulting in 373 eligible interventional trials. Each NCT identifier was then cross-checked to determine whether a corresponding publication existed. On this basis, a further PubMed search was conducted to link registered trials with published articles, identifying 68 trials with at least one publication for final analysis. The verification of the registered trials and the search and association in PubMed were all completed manually. The retrieval process is illustrated in Fig. 1.

### Data extraction

To avoid issues such as text misalignment and character encoding errors when downloading large volumes of data into Excel, we accessed the official ClinicalTrials.gov API (https://clinicaltrials.gov/api/int/studies) and applied specific parameters to retrieve and filter relevant study records. Key variables extracted included: temporal characteristics (registration date, start date, first submit date), geographic distribution (countries, regions, number of sites), study design (study type, study status, condition, phase, randomization, masking, allocation, primary purpose, primary outcome measures), demographic characteristics (age groups, sex, enrollment targets) and funder type.

Study type was categorized as Interventional or Observational, with the Interventional category also including expanded access studies. Study status was classified as Completed, Not completed, Ongoing, or Other. Completed trials included those finished and approved for marketing. Not completed referred to trials that were suspended, terminated, or withdrawn. Ongoing included those enrolling by invitation, not yet recruiting, recruiting, or active but not recruiting. Other included trials with unknown or unavailable status. Trial phases were grouped as Phase 1–2, Phase 2–3, and Phase 3–4. Phase 1–2 included early Phase 1, Phase 1, and combined Phase 1/2 studies; Phase 2–3 included Phase 2 and combined Phase 2/3 studies; and Phase 3–4 included Phase 3 and Phase 4 studies. Primary purpose was categorized as Treatment or Other, with the latter encompassing device feasibility, diagnostic, prevention, screening, and health services research. Funding source was grouped as Industry, NIH or other government, and Other (including networks and non-governmental sponsors). Geographic regions were classified as North America, Asia, and Other, with the latter including Europe and Oceania. Primary outcomes were categorized based on the specific endpoints reported; each outcome was counted individually, meaning a trial could contribute more than once if it reported multiple outcomes.

### Statistical analysis

The final dataset was exported to Excel in structured format, with all statistical analyses performed using SAS 9.4. Descriptive statistics were calculated for all variables, with categorical variables presented as frequencies and percentages. Comparative analyses between liver cancer and non-liver cancer studies were conducted using chi-square tests for categorical variables, with statistical significance set at p < 0.05. Given the descriptive and exploratory nature of this registry-based analysis, multivariable modeling was not performed, as the primary aim was to summarize trial characteristics rather than to identify independent predictors. A word cloud was generated from clinical trial titles using Python (wordcloud package) to visualize the distribution of disease areas and interventions.

## Results

### Overview of Yttrium-90 clinical trial characteristics

A total of 373 clinical trials were identified (Table 1). The majority were interventional (86.9%) and primarily treatment-focused (93.1%). Most were single-center (70.1%) with small sample sizes: 54.2% enrolled ≤ 30 participants, while only 14.8% enrolled more than 100. Pediatric inclusion was rare (6.4%). Nearly half of the trials were Phase 1–2 (47.7%) or Phase 2–3 (45.8%), with only 6.5% reaching Phase 3–4. Randomization was reported in 43.5% of trials, but masking was rare (3.4%). Most studies were funded by non-industry, non-government sources (82.8%), with limited support from industry (13.4%) or NIH/government (3.8%).

**Table 1:** Characteristics of Yttrium-90 related clinical trials comparing liver cancer and non-liver cancer studies

	No/Total.No (%)			
Characteristic	Liver cancer	Non-liver cancer	Total	*P* Value
**Study type**				< 0.001
Interventional	128/166 (77.1)	196/207 (94.7)	324/373 (86.9)	
Observational	38/166 (22.9)	11/207 (5.3)	49/373 (13.1)	
**Study status**				< 0.001
Completed	59/166 (35.5)	106/207 (51.2)	165/373 (44.2)	
Not completed	34/166 (20.5)	59/207 (28.5)	93/373 (25.0)	
Ongoing	62/166 (37.3)	18/207 (8.7)	80/373 (21.4)	
Others	11/166 (6.6)	24/207 (11.6)	35/373 (9.4)	
**Phases**				0.040
Phase1-2	33/90 (36.7)	99/187 (52.9)	132/277 (47.7)	
Phase2-3	50/90 (55.6)	77/187 (41.2)	127/277 (45.8)	
Phase3-4	7/90 (7.8)	11/187 (5.9)	18/277 (6.5)	
**Primary purpose**				< 0.001
Treatment	107/125 (85.6)	192/196 (98.0)	299/321 (93.1)	
Others	18/125 (14.4)	4/196 (2.0)	22/321 (6.9)	
**Sex**				0.016
All	166/166 (100)	200/207 (96.6)	366/373 (98.1)	
Male	0	3/207 (1.4)	3/373 (0.8)	
Female	0	4/207 (1.9)	4/373 (1.1)	
**Age**				0.016
Include child	5/166 (3.0)	19/207 (9.2)	24/373 (6.4)	
No child	161/166 (97.0)	188/207 (90.8)	349/373 (93.6)	
**Enrollment**				< 0.001
1–30	72/151 (47.7)	107/179 (59.8)	179/330 (54.2)	
31–100	50/151 (33.1)	52/179 (29.1)	102/330 (30.9)	
> 100	29/151 (19.2)	20/179 (11.2)	49/330 (14.8)	
**Allocation**				< 0.001
Randomized	30/42 (71.4)	20/73 (27.4)	50/115 (43.5)	
Non randomized	12/42 (28.6)	53/73 (72.6)	65/115 (56.5)	
**Masking**				
No	115/122 (94.3)	173/176 (98.3)	288/298 (96.6)	
Yes	7/122 (5.7)	3/176 (1.7)	10/298 (3.4)	
**Funder type**				< 0.001
Industry	17/166 (10.2)	33/207 (15.9)	50/373 (13.4)	
NIH or other gov	2/166 (1.2)	12/207 (5.8)	14/373 (3.8)	
Others	147/166 (88.6)	162/207 (78.3)	309/373 (82.8)	
**Region**				
North America	103/166 (62.1)	152/207 (73.4)	255/373 (68.4)	
Asia	33/166 (19.9)	10/207 (4.8)	43/373 (11.5)	
Others	30/166 (18.1)	45/207 (21.7)	75/373 (20.1)	
**Number of locations**				0.002
1	121/154 (78.6)	123/194 (63.4)	244/348 (70.1)	
> 1	33/154 (21.4)	71/194 (36.6)	104/348 (29.9)	

Liver cancer includes HCC, ICC and metastatic liver cancer. Among the study type, ‘Interventional’ includes interventional and expanded access. Among the study status, ‘Completed’ includes completed and approved for marketing; ‘Not completed’ includes suspended, terminated and withdrawn; ‘Ongoing’ included enrolling by invitation, active not recruiting, not yet recruiting and recruiting; and ‘Others’ includes unknown and no longer available. Among the phases, phase1-2 includes early phase1, phase1 and ‘phase1, phase2’; phase2-3 includes phase2 and ‘phase2, phase3’; phase3-4 includes phase3 and phase4. Among primary purpose, ‘Others’ includes device feasibility, diagnostic, health services research, prevention, screening and other primary purposes. Among funder type, ‘Others’ includes net work and other funders. Among region, ‘Others’ includes Europe and Oceania

**Fig. 2:**
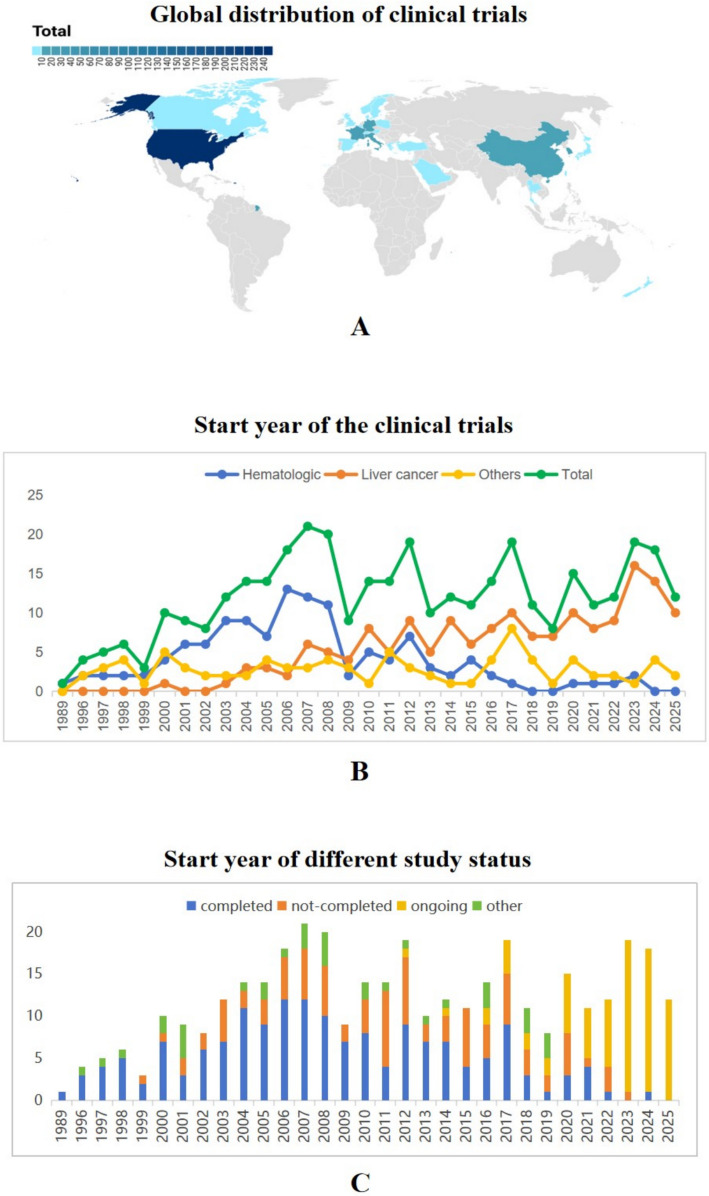
Characteristics of Yttrium-90 related clinical trials. **A** Global geographic distribution of trials by number; **B** Temporal trends in the initiation of clinical trials. Hematologic includes Lymphoma and Leukemia. Liver cancer includes HCC, ICC and metastatic liver cancer; **C** Study start year stratified by study status: completed includes completed and approved for marketing; not-completed includes suspended, terminated and withdrawn; ongoing included enrolling by invitation, active not recruiting, not yet recruiting and recruiting; and other includes unknown and no longer available

Geographically, trials were concentrated in North America (68.4%), particularly the United States (66%), followed by Europe (19.8%), Asia (11.5%), and Oceania (0.3%) (Table 1). At the country level, the top contributors were the United States (n = 246), China (n = 22), France (n = 21), Italy (n = 15), and Germany (n = 11) (Fig. 2A; Table S1). Temporal analysis showed that early trials in the 1980s–1990s primarily addressed hematologic malignancies, but after 2000, liver cancer became the dominant indication, with subsequent expansion into other tumors (Fig. 2B; Table S2). Since 2018, most newly initiated studies were categorized as not completed, ongoing, or other (Fig. 2C; Table S3).

### Comparisons between liver cancer and non-liver cancer studies

Marked differences emerged between liver cancer (n = 166) and non-liver cancer (n = 207) trials (Table 1). Liver cancer trials were more likely to be observational (22.9% vs. 5.3%, p < 0.001), ongoing at the time of the analysis (37.3% vs. 8.7%, p < 0.001), in advanced phases (phases 2 and above: 63.4% vs. 47.1%), and randomized (71.4% vs. 27.4%). They were also more commonly conducted in Asia (19.9% vs. 4.8%) and predominantly single-center (78.6% vs. 63.4%). In contrast, non-liver cancer studies were more frequently funded by industry or government (21.7% vs. 11.4%).

When restricted to interventional trials (Table S4), these trends persisted: liver cancer trials were more likely to be ongoing (39.8% vs. 7.7%), randomized (71.4% vs. 27.4%) and more commonly conducted in Asia (21.1% vs. 2.6%). Among completed trials (Table S5), liver cancer studies more often included phase 3–4 designs (12.5% vs. 4.3%) and randomized methodologies (76.9% vs. 14.3%), and were also more likely to be conducted in Asia (13.8% vs. 4.7%), compared with non-liver cancer studies.

### Indications of Yttrium-90 clinical trials

**Fig. 3:**
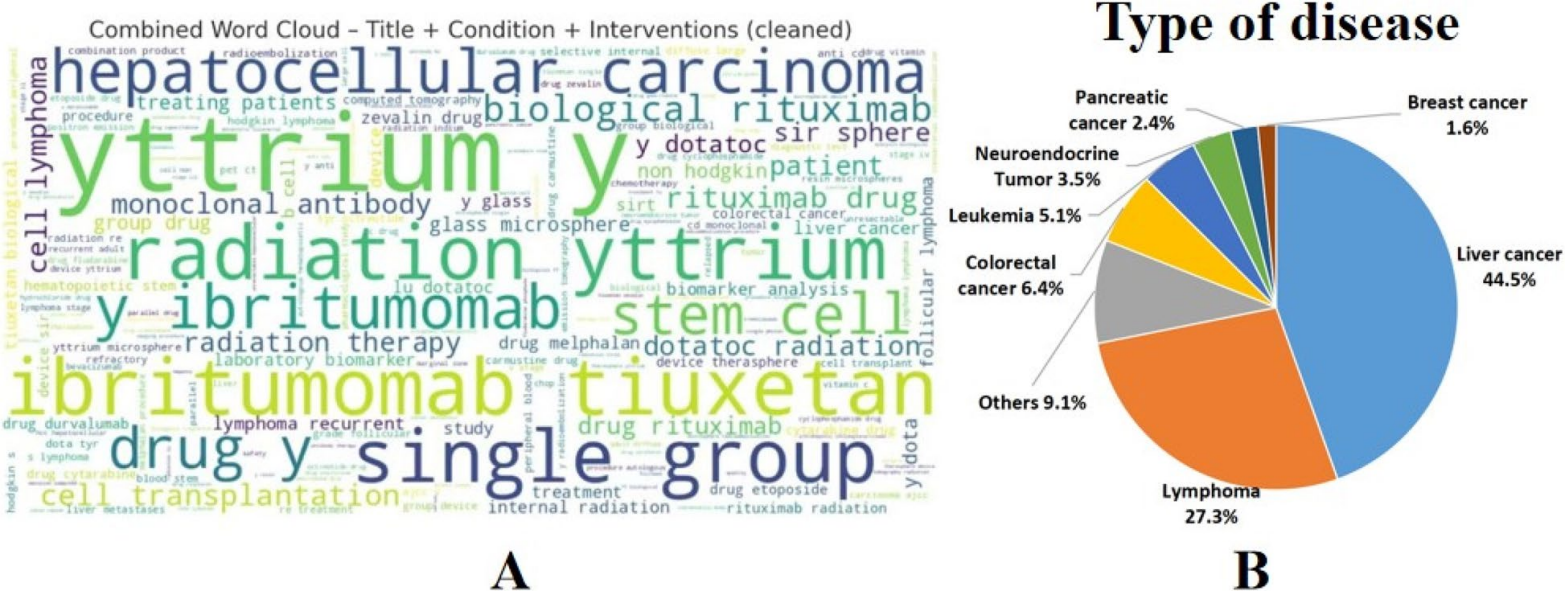
Word cloud of clinical trial titles, conditions, and interventions combined and distribution of diseases. **A** Word cloud of clinical trial titles, conditions, and interventions combined. The Y-90 trials mainly focused on the fields of liver cancer and lymphoma. Among them, there were more trials involving intervention measures such as radioembolization, ibutimodilimab, and rituximab. **B** Distribution of diseases. Liver cancer includes HCC, ICC and metastatic liver cancer. Liver cancer accounted for 44.5%, lymphoma accounted for 27.3%. The rest were colorectal cancer metastasis (6.4%), leukemia (5.1%), neuroendocrine tumors (3.5%), pancreatic cancer (2.4%), breast cancer (1.6%), and other malignant tumors (9.1%)

The word cloud (Fig. 3A) shows that Y-90 trials are heavily concentrated in HCC and lymphoma, split between device-based (radioembolization) and antibody-based (ibritumomab tiuxetan, rituximab) interventions. It also reveals a predominance of single-group designs and experimental expansions into areas like stem cell transplantation.

As shown in Fig. 3B, nearly three-quarters of all Y-90 trials involved liver cancer (44.5%) or lymphoma (27.3%), confirming these as the core indications. Smaller proportions studied colorectal cancer metastases (6.4%), leukemia (5.1%), neuroendocrine tumors (3.5%), pancreatic cancer (2.4%), breast cancer (1.6%), and other malignancies (9.1%).

### Reported outcomes

Table 2 presents outcomes by trails phases. In Phase 1–2 studies, safety endpoints dominated, including adverse events/toxicity (45.4%), maximum tolerated dose (38.9%), and dose-limiting toxicities (18.5%). Early-phase trials also occasionally assessed survival-related outcomes such as overall survival (OS), progression-free survival (PFS), complete response (CR), time to progression (TTP), and disease-free survival (DFS). Phase 2–3 trials shifted focus toward efficacy, with PFS (22.8%) as the most frequently reported outcome, followed by adverse events (18.7%), CR (13.8%), and overall or objective response rates (11–13%). In Phase 3–4 trials, survival endpoints predominanted, with OS (37.5%), PFS (25.0%), and TTP (25.0%) most frequently reported, although 18.8% of trails continued to prioritized safety. For trials listing multiple primary endpoints, each endpoint was counted separately; thus, a study could contribute more than once if more than one primary outcome was specified.

**Table 2:** Primary outcomes reported across different phases of Yttrium-90 related clinical trials

Phase	Primary Outcome	Count/Total.count(%)
Phase1-2	Adverse events/safety/toxicity	49/108 (45.4)
	Maximum tolerated dose (MTD)	42/108 (38.9)
	Dose limiting toxicities (DLT)	20/108 (18.5)
	Survival time/overall survival	6/108 (5.6)
	Progression free survival (PFS)	6/108 (5.6)
	Complete response (CR)	4/108 (3.7)
	Biodistribution	4/108 (3.7)
	Objective response rate	4/108 (3.7)
	Tumor response	4/108 (3.7)
	Time to progression (TTP)	3/108 (2.8)
	Overall response rate	3/108 (2.8)
	Pharmacokinetics and tumor targeting	2/108 (1.9)
	Disease free survival	1/108 (0.9)
Phase2-3	Progression free survival (PFS)	28/123 (22.8)
	Adverse events/safety/toxicity	23/123 (18.7)
	Complete response rate (CR)	17/123 (13.8)
	Objective response rate (ORR)	16/123 (13.0)
	Overall response rate (ORR)	14/123 (11.4)
	Overall survival/Survival time	10/123 (8.1)
	Event free survival (EFS)	7/123 (5.7)
	Tumor response	6/123 (4.9)
	Time to progression (TTP)	4/123 (3.3)
	Duration of response	2/123 (1.6)
	Treatment related mortality (TRM)	2/123 (1.6)
	Maximum tolerated dose (MTD)	1/123 (0.8)
	Disease control rate (DCR)	1/123 (0.8)
	Time to treatment failure (TTF)	1/123 (0.8)
Phase3-4	Overall survival/survival time	6/16 (37.5)
	Progression free survival (PFS)	4/16 (25.0)
	Time to progression (TTP)	4/16 (25.0)
	Safety/toxicity	3/16 (18.8)
	Complete response (CR)	1/16 (6.3)
	Quality of life	1/16 (6.3)

Among the phases, phase1-2 includes early phase1, phase1 and ‘phase1, phase2’; phase2-3 includes phase2 and ‘phase2, phase3’; phase3-4 includes phase3 and phase4

### Reasons for trial discontinuation

Among the 93 trials terminated, suspended, or withdrawn, the leading reason was accrual difficulties (38.7%) (Table 3). Other frequent causes included lack of funding (7.5%) and sponsor withdrawal (6.5%). Additional reasons were drug or device supply issues (4.3%), interim analyses not meeting predefined criteria (3.2%), principal investigator departure (3.2%), and practice changes (2.1%). The COVID-19 pandemic was cited in 2.1% of cases. Notably, 16.1% provided no reason, underscoring gaps in registry transparency.

**Table 3:** Reason of not completed

Reason of not completed	Count/Total.count(%)
Accrual issues	36/93 (38.7)
Lack of funding	7/93 (7.5)
Withdrawn by the study sponsor or company	6/93 (6.5)
Drug supply unavailable	3/93 (3.2)
The results of the interim analysis did not meet the pre-set criteria	3/93 (3.2)
PI left the institution	3/93 (3.2)
Changes in practice	2/93 (2.1)
Pandemic	2/93 (2.1)
Product supply unavailable	1/93 (1.1)
Unavailability of study device	1/93 (1.1)
Business reasons	1/93 (1.1)
Research Cancelled	1/93 (1.1)
Not feasible at this time	1/93 (1.1)
Participants are no longer being examined or treated	1/93 (1.1)
Record created in error—not a research study	1/93 (1.1)
Replaced with another study	1/93 (1.1)
This project has been updated and resubmitted	1/93 (1.1)
Trial completed prematurely	1/93 (1.1)
DSMC—directed closure	1/93 (1.1)
Administratively complete	1/93 (1.1)
New device was approved by FDA	1/93 (1.1)
Completed recruitment of patients on 15 Dec. 2010	1/93 (1.1)
Off—study clinical use of Y-90 glass microspheres	1/93 (1.1)
1st level of sequential inclusion reached	1/93 (1.1)
No reason was given	15/93 (16.1)

### Trials with published results

We further investigated trials that had published results (Table 4 and Table S6). Of the 373 eligible studies, 68 (18.2%) reported at least one publication, including 35 focused on liver cancer and 33 on non-liver cancer indications. Most produced only a single publication (64.7%), while 23.5% generated two and 11.8% generated three or more. Publication patterns were similar between liver cancer and non–liver cancer studies. Nearly half of these studies were conducted at a single center (42.7%), one-third were multicenter (35.3%), and 22.6% did not specify the setting.

**Table 4:** Characteristics of trials based on the published article

		No Total No(%)	
Characteristic	Liver cancer	Non-liver cancer	Total
**Number of publications**			
1	21/35 (60.0)	23/33 (69.7)	44/68 (64.7)
2	8/35 (22.6)	8/33 (24.2)	16/68 (23.4)
≥ 3	6/35 (17.1)	2/33 (6.0)	8/68 (11.8)
**Number of locations**			
1	14/35 (40.0)	15/33 (45.5)	29/68 (42.7)
> 1	15/35 (42.6)	2/33 (6.1)	17/68 (25.0)
NA	6/35 (17.1)	16/33 (48.5)	22/68 (32.4)
**Enrollment**			
1–30	14/35 (40.0)	12/33 (36.4)	26/68 (38.2)
31–100	8/35 (22.9)	13/33 (39.4)	21/68 (30.9)
> 100	9/35 (25.7)	4/33 (12.1)	13/68 (19.1)
NA	4/35 (11.4)	4/33 (12.1)	8/68 (11.8)
**Median age**			
≤ 18	0	0	0
> 18	22/35 (62.9)	25/33 (75.8)	47/68 (69.1)
NA	13/35 (37.1)	8/33 (24.2)	15/68 (22.1)
**Median overall survival**			
0–12 months	4/35 (11.4)	1/33 (3.0)	5/68 (7.4)
13–24 months	7/35 (20.0)	2/33 (6.0)	9/68 (13.2)
> 24 months	7/35 (20.0)	2/33 (6.0)	9/68 (13.2)
NA	17/35 (48.6)	28/33 (84.8)	45/68 (66.1)
**Median progression-free survival**			
0–12 months	15/35 (42.9)	1/33 (3.0)	16/68 (23.5)
13–24 months	0	1/33 (3.0)	1/68 (1.5)
> 24 months	3/35 (8.6)	5/33 (15.1)	8/68 (11.7)
NA	17/35 (48.6)	26/33 (78.8)	25/68 (36.8)
**Median follow-up**			
0–12 months	2/35 (5.7)	0	2/68 (2.9)
13–24 months	3/35 (8.6)	0	3/68 (4.4)
> 24 months	4/35 (11.4)	4/33 (12.1)	8/68 (11.8)
NA	26/35 (74.3)	29/33 (87.9)	55/68 (80.1)
**Objective response rate**			
≤ 50%	11/35 (31.4)	2/33 (6.0)	12/68 (17.6)
> 50%	6/35 (17.1)	8/33 (24.2)	14/68 (20.6)
NA	18/35 (51.4)	23/33 (69.7)	41/68 (60.3)
**Complete response**			
≤ 50%	7/35 (20.0)	5/33 (15.1)	12/68 (17.6)
> 50%	0	3/33 (9.1)	3/68 (4.4)
NA	28/35 (80.0)	24/33 (72.7)	52/68 (76.5)
**Partial response**			
≤ 50%	8/35 (22.9)	5/33 (15.2)	13/68 (19.1)
> 50%	1/35 (2.9)	3/33 (9.1)	4/68 (5.9)
NA	26/35 (74.3)	25/33 (75.8)	51/68 (75.0)
**Stable disease**			
≤ 50%	5/35 (14.3)	3/33 (9.1)	8/68 (11.8)
> 50%	1/35 (2.9)	1/33 (3.0)	2/68 (2.9)
NA	29/35 (82.9)	29/33 (87.9)	58/68 (85.3)
**Disease control rate**			
≤ 50%	0	1/33 (3.0)	1/68 (1.5)
> 50%	11/35 (31.4)	3/33 (9.1)	14/33 (42.4)
NA	24/35 (68.6)	29/33 (87.9)	53/68 (78.0)
**Non-relapse mortality**			
≤ 50%	1/35 (2.9)	3/33 (9.1)	4/68 (5.9)
> 50%	4/35 (11.4)	0	4/68 (5.9)
NA	30/35 (85.7)	30/33 (91.0)	60/68 (88.2)
**Adverse events (grade ≥ 3)**			
≤ 50%	14/35 (40.0)	1/33 (3.0)	15/68 (22.1)
> 50%	3/35 (8.6)	3/33 (9.1)	6/68 (8.9)
NA	18/35 (51.4)	29/33 (87.9)	47/68 (69.1)
**Serious adverse event**			
≤ 50%	7/35 (20.0)	1/33 (3.0)	8/68 (11.8)
> 50%	1/35 (2.9)	0	1/68 (1.5)
NA	27/35 (77.1)	32/33 (97.0)	59/68 (86.8)

Liver cancer includes HCC, ICC and metastatic liver cancer

Efficacy outcomes were more frequently reported in liver cancer studies. For median overall survival (mOS), 11.4%, 20.0%, and 20.0% of liver cancer trials documented mOS of 0–12 months, 13–24 months, and > 24 months, respectively, compared with only 3.0%, 6.0%, and 6.0% of non-liver cancer studies. Similar differences were observed for PFS, median follow-up (mFU), ORR, stable disease (SD), disease control rate (DCR), and non-relapse mortality (NRM), all of which were reported more consistently in liver cancer trials. Adverse event reporting also varied: grade ≥ 3 adverse events were reported in 48.6% of liver cancer trials versus 12.1% of non–liver cancer trials, and serious adverse events (SAEs) in 22.9% versus 3.0%, respectively.

## Discussion

This systematic registry analysis provides the first comprehensive overview of the global landscape of Y-90 clinical trials. Overall, Y-90 research has grown steadily since the early 2000 s, particularly in liver cancer. However, the evidence base remains characterized by small, early-phase, predominantly single-center studies, geographic concentration in North America, and limited result dissemination. Together, these findings indicate that while clinical interest in Y-90 continues to grow, the supporting research infrastructure and evidence maturity remain uneven. These patterns provide important context for interpreting the current role of Y-90 in oncology and inform priorities for future investigation.

### Expansion of Y-90 research and shifting indications

The steady growth of Y-90 research since the early 2000 s reflects its increasing recognition as a clinically relevant therapeutic modality. Initially developed for hematologic malignancies, Y-90 has progressively shifted toward solid tumors, with liver cancer now the predominant indication [6, 11, 12]. This transition aligns with the high global burden of liver cancer and the anatomical advantages of liver-directed therapy, where Y-90 can selectively target tumors while sparing normal parenchyma [2]. The rise of liver cancer related trials also highlights the need for effective alternatives to TACE and systemic therapy, particularly in patients who are ineligible for surgery or transplantation.

Radiolectomy, also known as ablation radioembolization, represents a targeted form of Y-90 therapy. This technique delivers high-dose radiation to two or fewer liver segments to achieve complete tumor inactivation while minimizing injury to adjacent parenchyma [13, 14]. Segmental resection has long been established as an effective therapy for HCC and radiolectomy has shown comparable efficacy in early-stage disease [13, 15]. In this analysis, three identified trials investigated segmentectomy approaches: NCT03248375, which evaluated TheraSphere-based segmentectomy in patients with unresectable HCC not eligible for thermal ablation under BCLC guidelines; NCT04235660, which compared radiolectomy with stereotactic body radiotherapy (SBRT) for early-stage (≤ 3 cm) HCC; and NCT06618300, a single-arm feasibility study assessing a simplified workflow for Y-90 radiolectomy in small, non-invasive HCCs (< 5 cm).

### Predominance of small, single-center, early-phase studies

Despite the expansion of Y-90 research, most trials remain small, single-center, and early- to mid-phase, with limited use of randomization or masking. These methodological constraints limit the ability to draw definitive conclusions regarding efficacy and safety and contribute to heterogeneity across the evidence base. While small, non-randomized studies are often more feasible in interventional oncology, they provide weaker evidence than adequately powered, multicenter randomized controlled trials (RCTs). Current guidelines reflect this evidence gap. In the Barcelona Clinic Liver Cancer (BCLC) staging system and the Korean Liver Cancer Association–National Cancer Center (KLCA-NCC) guidelines, TARE is recommended as an alternative treatment for early stage HCC [16]. Similarly, Asia–Pacific guidelines support SIRT as an alternative to TACE as first-line therapy for patients with intermediate or advanced HCC without extrahepatic disease [17]. However, the paucity of large phase 3–4 trials continues to limit the integration of Y-90 into international recommendations and highlights the urgent need for collaborative efforts to design and conduct more rigorous studies [18].

### Geographic imbalance in trial activity

The geographic imbalance observed in Y-90 research raises concerns about the generalizability of findings. Although North America accounted for more than two-thirds of all studies, Asia, where over 70% of global HCC cases occur, contributed only 11.5% of registered trials. This mismatch is striking given that incidence rates in East Asia are several-fold higher than in Western countries [19]. The underrepresentation of Asian populations may compromise the external validity of trial findings, particularly as differences in etiology (HBV vs. HCV or alcohol), liver function reserve, and healthcare access could influence both treatment response and toxicity [17]. Moreover, the lack of region-specific evidence limits the development of local guidelines and reimbursement policies, further restricting patient access to Y-90 therapy. Addressing this gap will require expanding trial infrastructure, encouraging industry and academic investment in high-burden regions, and fostering multinational collaborations to generate evidence relevant to diverse populations and healthcare systems.

### Limited efficiency and dissemination of trial results

Trial efficiency and dissemination remain major challenges. Nearly one-quarter of studies were discontinued, most often due to accrual difficulties, which are common in interventional oncology where patient eligibility can be restrictive and competing therapeutic options are available. The lack of transparent reporting of termination reasons further undermines confidence in the evidence base. Moreover, fewer than 20% of registered trials had published results, raising concerns about publication bias and the loss of valuable clinical data. Greater efforts are needed to improve patient recruitment strategies, mandate transparent reporting of trial discontinuation, and ensure timely dissemination of results, thereby maximizing the scientific and clinical value of Y-90 research.

### Clinical implications and future directions

Cost and accessibility further challenge its broader adoption [9]. For instance, a single Y-90 treatment in China costs approximately RMB 400,000 (USD ~ 56,000), while the average annual per capita disposable income in 2024 in China was RMB 41,314 (USD ~ 5,801) [20–22]. As Y-90 is not uniformly covered by national insurance systems, many patients face substantial out-of-pocket expenses [20, 21]. Beyond individual affordability, these economic considerations may influence healthcare reimbursement decisions and coverage policies [23]. In this context, the generation of high-quality clinical evidence, particularly from adequately powered trials and real-world cost-effectiveness analyses, will be essential to inform payer evaluations and support sustainable integration of Y-90 into routine practice.

The anticipated introduction of produced Y-90 microspheres (made in China) may reduce treatment costs [24], potentially improving access. However, broader reimbursement and adoption will depend not only on price reduction but also on rigorous validation of efficacy, safety, and value across diverse healthcare settings.

Emerging evidence also highlights the importance of personalized dosimetry. Studies demonstrate a clear dose, response relationship with Y-90 microspheres in the treatment of HCC and liver metastases [25, 26]. For instance, NCT04172714 established dose–response and dose–toxicity thresholds in patients receiving resin-based Y-90 therapy, while NCT02582034 showed that personalized dosimetry significantly improved objective response rates compared with standard dosing in locally advanced HCC. These findings suggest that individualized dose optimization may enhance therapeutic efficacy in clinical practice.

Looking forward, future research may benefit from prioritizing adequately powered multicenter RCTs, particularly in underrepresented regions such as Asia, and from further exploration of combination strategies integrating Y-90 with systemic therapies (e.g., immunotherapies and targeted agents). For instance, Y-90 in combination with immune checkpoint inhibitors, including the PD-1 inhibitor nivolumab and PD-L1–based regimens such as atezolizumab/bevacizumab or durvalumab, is currently under active clinical investigation [27]. Early-phase studies have reported encouraging signals of safety and potential efficacy, supporting continued exploration of these combination approaches [28, 29]. Such efforts could help to better define the optimal role of Y-90 in contemporary oncologic care.

### Limitations and strengths

This study has several limitations. First, it was restricted to ClinicalTrials.gov, and trials registered exclusively on other national or regional platforms may not have been captured. As a result, regional comparisons reflect trials recorded in this international registry rather than the absolute volume of global research activity. Second, registry entries are subject to reporting bias and incomplete information, particularly regarding outcomes and reasons for discontinuation. Third, linking NCT identifiers with publications may also have missed articles when trial numbers were not cited. In addition, outcome definitions varied substantially across individual trials, especially for response metrics, thereby limiting direct quantitative comparisons, which limits direct quantitative comparisons. Finally, the cross-sectional design captures trial status at one point in time and may not reflect subsequent updates or late publications.

Despite these limitations, the study offers a comprehensive and transparent overview of Y-90 clinical research worldwide. Use of the largest publicly accessible registry enabled standardized data collection and cross-disease comparisons, while linkage with PubMed strengthened the evaluation of result dissemination.

## Conclusions

Y-90 clinical research has expanded globally, with liver cancer emerging as the predominant focus. However, trials remain small, geographically concentrated, and poorly disseminated, with high discontinuation rates. Addressing cost barriers, improving methodological rigor, and ensuring transparent reporting will be essential to advance the field. Future multicenter trials, particularly in high-burden regions, and combination strategies with systemic therapies may help define the optimal role of Y-90 in contemporary oncologic care.

## Supplementary Information

Supplementary Material 1.

## Data Availability

The datasets used and/or analyzed during the current study are available from the corresponding author upon reasonable request.
