# Supplementary material for: Global landscape of yttrium-90 clinical trials: a systematic registry-based analysis
Source: Trials. 2026 Mar 27;27:355. doi: 10.1186/s13063-026-09659-7 (PMC13147879; doi:10.1186/s13063-026-09659-7)
Supplement: Supplementary file 1 — Supplementary Material 1. [file 13063_2026_9659_MOESM1_ESM.docx]

**Supplementary table 1: Distribution of the number of trials across global countries**

| **Country** | **Number(%)** |
| --- | --- |
| United States | 246 (66.0) |
| China | 22 (5.9) |
| France | 21 (5.6) |
| Italy | 15 (4.0) |
| Germany | 11 (2.9) |
| Korea | 10 (2.7) |
| Canada | 9 (2.4) |
| Belgium | 7 (1.9) |
| United Kingdom | 7 (1.9) |
| Spain | 4 (1.1) |
| Japan | 3 (0.8) |
| Singapore | 3 (0.8) |
| Norway | 2 (0.5) |
| Switzerland | 2 (0.5) |
| Brunei Darussalam | 1 (0.3) |
| Israel | 1 (0.3) |
| Saudi Arabia | 1 (0.3) |
| Thailand | 1 (0.3) |
| Turkey | 1 (0.3) |
| Austria | 1 (0.3) |
| Denmark | 1 (0.3) |
| Greece | 1 (0.3) |
| Poland | 1 (0.3) |
| Sweden | 1 (0.3) |
| New Zealand | 1 (0.3) |

Corresponding to Figure 2A.

**Supplementary table 2: Distribution of the number of clinical trials by disease category and start year**

|  | **Number** | | | |
| --- | --- | --- | --- | --- |
| **Start year** | **Hematologic** | **Liver cancer** | **Others** | **Total** |
| 1989 | 1 | 0 | 0 | 1 |
| 1996 | 2 | 0 | 2 | 4 |
| 1997 | 2 | 0 | 3 | 5 |
| 1998 | 2 | 0 | 4 | 6 |
| 1999 | 2 | 0 | 1 | 3 |
| 2000 | 4 | 1 | 5 | 10 |
| 2001 | 6 | 0 | 3 | 9 |
| 2002 | 6 | 0 | 2 | 8 |
| 2003 | 9 | 1 | 2 | 12 |
| 2004 | 9 | 3 | 2 | 14 |
| 2005 | 7 | 3 | 4 | 14 |
| 2006 | 13 | 2 | 3 | 18 |
| 2007 | 12 | 6 | 3 | 21 |
| 2008 | 11 | 5 | 4 | 20 |
| 2009 | 2 | 4 | 3 | 9 |
| 2010 | 5 | 8 | 1 | 14 |
| 2011 | 4 | 5 | 5 | 14 |
| 2012 | 7 | 9 | 3 | 19 |
| 2013 | 3 | 5 | 2 | 10 |
| 2014 | 2 | 9 | 1 | 12 |
| 2015 | 4 | 6 | 1 | 11 |
| 2016 | 2 | 8 | 4 | 14 |
| 2017 | 1 | 10 | 8 | 19 |
| 2018 | 0 | 7 | 4 | 11 |
| 2019 | 0 | 7 | 1 | 8 |
| 2020 | 1 | 10 | 4 | 15 |
| 2021 | 1 | 8 | 2 | 11 |
| 2022 | 1 | 9 | 2 | 12 |
| 2023 | 2 | 16 | 1 | 19 |
| 2024 | 0 | 14 | 4 | 18 |
| 2025 | 0 | 10 | 2 | 12 |

Corresponding to Figure 2B. Hematologic includes Lymphoma and Leukemia; Liver cancer includes HCC, ICC and metastatic liver cancer; others includes colorectal cancer, neuroendocrine tumor, pancreatic cancer, breast cancer and other diseases.

**Supplementary table 3: Distribution of the number of clinical trials by study status and start year**

|  | **Number** | | | | |
| --- | --- | --- | --- | --- | --- |
| **Start year** | **Completed** | **Not-completed** | **Ongoing** | **Others** | **Total** |
| 1989 | 1 | 0 | 0 | 0 | 1 |
| 1996 | 3 | 0 | 0 | 1 | 4 |
| 1997 | 4 | 0 | 0 | 1 | 5 |
| 1998 | 5 | 0 | 0 | 1 | 6 |
| 1999 | 2 | 1 | 0 | 0 | 3 |
| 2000 | 7 | 1 | 0 | 2 | 10 |
| 2001 | 3 | 2 | 0 | 4 | 9 |
| 2002 | 6 | 2 | 0 | 0 | 8 |
| 2003 | 7 | 5 | 0 | 0 | 12 |
| 2004 | 11 | 2 | 0 | 1 | 14 |
| 2005 | 9 | 3 | 0 | 2 | 14 |
| 2006 | 12 | 5 | 0 | 1 | 18 |
| 2007 | 12 | 6 | 0 | 3 | 21 |
| 2008 | 10 | 6 | 0 | 4 | 20 |
| 2009 | 7 | 2 | 0 | 0 | 9 |
| 2010 | 8 | 4 | 0 | 2 | 14 |
| 2011 | 4 | 9 | 0 | 1 | 14 |
| 2012 | 9 | 8 | 1 | 1 | 19 |
| 2013 | 7 | 2 | 0 | 1 | 10 |
| 2014 | 7 | 3 | 1 | 1 | 12 |
| 2015 | 4 | 7 | 0 | 0 | 11 |
| 2016 | 5 | 4 | 2 | 3 | 14 |
| 2017 | 9 | 6 | 4 | 0 | 19 |
| 2018 | 3 | 3 | 2 | 3 | 11 |
| 2019 | 1 | 2 | 2 | 3 | 8 |
| 2020 | 3 | 5 | 7 | 0 | 15 |
| 2021 | 4 | 1 | 6 | 0 | 11 |
| 2022 | 1 | 3 | 8 | 0 | 12 |
| 2023 | 0 | 1 | 18 | 0 | 19 |
| 2024 | 1 | 0 | 17 | 0 | 18 |
| 2025 | 0 | 0 | 12 | 0 | 12 |

Corresponding to Figure 2C. Completed includes completed and approved for marketing; not-completed includes suspended, terminated and withdrawn; ongoing included enrolling by invitation, active not recruiting, not yet recruiting and recruiting; and others includes unknown and no longer available.

**Supplementary table 4: Characteristics of Yttrium-90 related interventional clinical trials comparing liver cancer and non-liver cancer studies**

|  | No/Total.No (%) | |
| --- | --- | --- |
| Characteristic | Liver cancer | Non-Liver cancer |
| **Study status** |  |  |
| Completed | 40/128 (31.3) | 99/196 (50.5) |
| Not completed | 30/128 (23.4) | 59/196 (30.1) |
| Ongoing | 51/128 (39.8) | 15/196 (7.7) |
| Others | 7/128 (5.5) | 23/196 (11.7) |
| **Phases** |  |  |
| Phase1-2 | 33/90 (36.7) | 99/187 (52.9) |
| Phase2-3 | 50/90 (55.6) | 77/187 (41.2) |
| Phase3-4 | 7/90 (7.8) | 11/187 (5.9) |
| **Primary purpose** |  |  |
| Treatment | 107/125 (85.6) | 192/196 (98.0) |
| Others | 18/125 (14.4) | 4/196 (2.0) |
| **Sex** |  |  |
| All | 128/128 (100) | 190/196 (96.9) |
| Male | 0 | 2/196 (1.0) |
| Female | 0 | 4/196 (2.0) |
| **Age** |  |  |
| Include child | 4/128 (3.1) | 17/196 (8.7) |
| No child | 124/128 (96.9) | 179/196 (91.3) |
| **Enrollment** |  |  |
| 1--30 | 58/114 (50.9) | 104/168 (61.9) |
| 31--100 | 41/114 (36.0) | 49/168 (29.2) |
| >100 | 15/114 (13.2) | 15/168 (8.9) |
| **Allocation** |  |  |
| Randomized | 30/42 (71.4) | 20/73 (27.4) |
| Non randomized | 12/42 (28.6) | 53/73 (72.6) |
| **Masking** |  |  |
| No | 115/122 (94.3) | 173/176 (98.3) |
| Yes | 7/122 (5.7) | 3/176 (1.7) |
| **Interventional model** |  |  |
| parallel | 32/123 (26.0) | 28/170 (16.5) |
| sequential | 91/123 (74.0) | 142/170 (83.5) |
| **Funder type** |  |  |
| Industry | 12/128 (9.4) | 29/196 (14.8) |
| NIH or other gov | 1/128 (0.8) | 12/196 (6.1) |
| Others | 115/128 (89.8) | 155/196 (79.1) |
| **Region** |  |  |
| North America | 82/128 (64.1) | 150/196 (76.5) |
| Asia | 27/128 (21.1) | 5/196 (2.6) |
| Others | 19/128 (14.8) | 41/196 (20.9) |
| **Number of locations** |  |  |
| 1 | 93/119 (78.2) | 115/184 (62.5) |
| >1 | 26/119 (21.8) | 69/184 (37.5) |

Liver cancer includes HCC, ICC and metastatic liver cancer. Among the study status, ‘Completed’ includes completed and approved for marketing; ‘Not completed’ includes suspended, terminated and withdrawn; ‘Ongoing’ included enrolling by invitation, active not recruiting, not yet recruiting and recruiting; and ‘Others’ includes unknown and no longer available.. Among the phases, phase1-2 includes early phase1, phase1 and ‘phase1, phase2’; phase2-3 includes phase2 and ‘phase2, phase3’; phase3-4 includes phase3 and phase4. Among primary purpose, ‘Others’ includes device feasibility, diagnostic, health services research, prevention, screening and other primary purposes. Among interventional model, ‘Sequential’ includes sequential and single group. Among funder type, ‘Others’ includes net work and other funders. Among region, ‘Others’ includes Europe and Oceania.

**Supplementary table 5: Characteristics of completed Yttrium-90 related clinical trials comparing liver cancer and non-liver cancer Studies**

|  | No/Total.No(%) | |
| --- | --- | --- |
| Characteristic | Liver cancer | Non-liver cancer |
| **Study type** |  |  |
| Interventional | 39/58 (67.2) | 99/106 (93.4) |
| Observational | 19/58 (32.8) | 7/106 (6.6) |
| **Sex** |  |  |
| All | 58/58 (100) | 103/106 (97.2) |
| Male | 0 | 0 |
| Female | 0 | 3/106 (2.8) |
| **Age** |  |  |
| Include child | 4/58 (6.9) | 13/106 (12.6) |
| No child | 54/58 (93.1) | 93/106 (87.7) |
| **Enrollment** |  |  |
| 1--30 | 23/57 (40.4) | 52/100 (53.0) |
| 31--100 | 18/57 (31.6) | 36/100 (36.0) |
| >100 | 16/57 (28.1) | 12/100 (12.0) |
| **Phases** |  |  |
| Phase1-2 | 10/24 (41.7) | 50/94 (53.2) |
| Phase2-3 | 11/24 (45.8) | 40/94 (42.6) |
| Phase3-4 | 3/24 (12.5) | 4/94 (4.3) |
| **Primary purpose** |  |  |
| Treatment | 34/39 (87.2) | 97/99 (98.0) |
| Others | 5/39 (12.8) | 2/99 (2.0) |
| **Funder type** |  |  |
| Industry | 4/58 (6.9) | 15/106 (14.2) |
| NIH or Other gov | 1/58 (1.7) | 8/106 (7.5) |
| Others | 53/58 (91.4) | 83/106 (78.3) |
| **Region** |  |  |
| North America | 37/58 (63.8) | 81/106 (76.4) |
| Asia | 8/58 (13.8) | 5/106 (4.7) |
| Others | 13/58 (22.4) | 20/106 (18.9) |
| **Number of locations** |  |  |
| 1 | 43/54 (79.6) | 62/101 (61.4) |
| >1 | 11/54 (20.4) | 39/101 (38.6) |
| **Allocation** |  |  |
| Randomized | 10/13 (76.9) | 5/35 (14.3) |
| Non randomized | 3/13 (23.1) | 30/35 (85.7) |
| **Masking** |  |  |
| No | 37/38 (97.4) | 89/89 (100) |
| Yes | 1/38 (2.6) | 0 |
| **Interventional model** |  |  |
| Parallel | 9/38 (23.7) | 11/88 (12.5) |
| Sequential | 29/38 (76.3) | 77/88 (87.5) |

Liver cancer includes HCC, ICC and metastatic liver cancer. Among the phases, phase1-2 includes early phase1, phase1 and ‘phase1, phase2’; phase2-3 includes phase2 and ‘phase2, phase3’; phase3-4 includes phase3 and phase4. Among primary purpose, ‘Others’ includes diagnostic, screening and other primary purposes. Among funder type, ‘Others’ includes net work and other funders. Among region, ‘Others’ includes Europe and Oceania. Among interventional model, ‘Sequential’ includes sequential and single group.

**Supplementary table 6: Characteristics of trials based on study protocols registered on ClinicalTrials.gov (NCT)**

| Characteristic | Liver cancer | No Total No(%)  Non-liver cancer | Total |
| --- | --- | --- | --- |
| **Study type** |  |  |  |
| Interventional | 25/35 (71.4) | 31/33 (93.9) | 56/68 (82.4) |
| Observation | 10/35 (28.6) | 2/33 (6.1) | 12/68 (17.6) |
| **Study status** |  |  |  |
| Completed | 24/35 (68.6) | 21/33 (63.6) | 45/68 (66.2) |
| Not completed | 3/35 (8.6) | 3/33 (9.1) | 6/68 (8.8) |
| Ongoing | 5/35 (14.3) | 6/33 (18.2) | 11/68 (16.2) |
| Others | 3/35 (8.6) | 3/33 (9.1) | 6/68 (8.8) |
| **Phases** |  |  |  |
| Phase 1-2 | 16/35 (45.7) | 29/33 (88.8) | 45/68 (66.2) |
| Phase 2-3 | 15/35 (33.3) | 17/33 (51.5) | 32/68 (47.1) |
| Phase 3-4 | 2/35 (5.7) | 0 | 2/68 (2.9) |
| **Primary purpose** |  |  |  |
| Treatment | 24/35 (68.6) | 31/33 (94.0) | 55/68 (80.1) |
| Others | 11/35 (31.5) | 2/33 (6.1) | 13/68 (19.1) |
| **Sex** |  |  |  |
| ALL | 35/35 (100.0) | 32/33 (97.0) | 67/68 (98.5) |
| Male | 0 | 1/33 (3.0) | 1/68 (1.5) |
| Female | 0 | 0 | 0 |
| **Age** |  |  |  |
| Include child | 1/35 (2.9) | 1/33 (3.0) | 2/68 (3.0) |
| No child | 34/35 (97.1) | 32/33 (97.0) | 66/68 (97.0) |
| **Enrollment** |  |  |  |
| 1-30 | 10/35 (28.6) | 13/33 (39.4) | 23/68 (33.8) |
| 31-100 | 15/35 (42.6) | 13/33 (39.4) | 28/68 (41.2) |
| ＞100 | 9/35 (25.7) | 6/33 (18.2) | 15/68 (22.1) |
| NA | 1/35 (2.6) | 1/33 (3.0) | 2/68 (3.0) |
| **Number of locations** |  |  |  |
| 1 | 19/35 (25.7) | 24/33 (72.7) | 43/68 (63.2) |
| ＞1 | 14/35 (40.0) | 8/33 (24.2) | 22/68 (32.4) |
| NA | 2/35 (5.7) | 1/33 (3.0) | 3/68 (4.4) |
| **Allocation** |  |  |  |
| Randomized | 7/35 (20.0) | 3/33 (9.1) | 10/68 (14.7) |
| Non randomized | 1/35 (2.9) | 8/33 (24.2) | 9/68 (13.2) |
| NA | 27/35 (77.1) | 22/33 (66.7) | 49/68 (72.1) |
| **Masking** |  |  |  |
| No | 25/35 (71.4) | 31/33 (93.9) | 56/68 (82.4) |
| None | 10/35 (28.6) | 2/33 (6.1) | 12/68 (17.6) |
| **Funder type** |  |  |  |
| Industry | 3/35 (8.6) | 2/33 (6.1) | 5/68 (7.4) |
| Network | 0 | 1/33 (3.0) | 1/68 (1.5) |
| others | 32/35 (91.4) | 30/33 (91.0) | 62/68 (91.2) |
| **Region** |  |  |  |
| North American | 5/35 (14.3) | 1/33 (3.0) | 6/68 (8.8) |
| Asia | 18/35 (51.4) | 17/33 (51.5) | 35/68 (51.5) |
| Others | 12/35 (34.3) | 15/33 (45.5) | 27/68 (39.7) |
| **Number Of Arm** |  |  |  |
| 0 | 6/35 (17.1) | 2/33 (6.1) | 8/68 (11.8) |
| 1 | 20/35 (5.7) | 23/33 (69.7) | 43/68 (63.2) |
| 2 | 8/35 (22.3) | 2/33 (6.1) | 10/68 (14.7) |
| ＞2 | 1/35 (2.9) | 6/33 (18.2) | 7/68 (10.3) |
| **Sponsor** |  |  |  |
| North America | 18/35 (51.4) | 17/33 (51.5) | 35/68 (51.5) |
| Europe | 11/35 (31.4) | 14/33 (40.0) | 25/68 (36.8) |
| Asia-Pacific | 5/35 (14.3) | 1/33 (3.0) | 6/68 (8.9) |
| Unknown | 1/35 (2.9) | 1/33 (3.0) | 2/68 (3.0) |

Liver cancer includes HCC, ICC and metastatic liver cancer. Among the study status completed includes completed and approved for marketing, not completed includes suspended, terminated and withdrawn, ongoing included enrolling by invitation, active not recruiting, not yet recruiting andrecruiting. Among the phases, phase 1-2 includes early phase l, phase l and ‘phase l, phase2’; phase 2-3 includes phase 2 and ‘phase 2, phase 3’; phase 3-4 includes phase 3 and phase 4. Under Funder type, ‘Others’ comprises government agencies, non-profit organizations, or any blended funding sources. Region indicates the geographic area or country where the study is actually conducted. The sponsor is the organization or individual that initiates, funds, and bears overall responsibility for the study.
